# Assessment of body dysmorphic disorder in patients with anorexia nervosa and bulimia nervosa. The final data of the study

**DOI:** 10.1192/j.eurpsy.2021.951

**Published:** 2021-08-13

**Authors:** E. Okonishnikova, A. Bryukhin, T. Lineva, I. Belokrylov

**Affiliations:** 1 Department Of Psychiatry And Medical Psychology, RUDN University Moscow., Moscow, Russian Federation; 2 Department Of Psychiatry And Medical Psychologi, Peoples Friendship University of Russia (RUDN University), Moscow, Russian Federation

**Keywords:** eating disorder, body dysmorphic disorder

## Abstract

**Introduction:**

Anorexia nervosa (AN) and bulimia nervosa (BN) occur predominantly females, take one of the first places in the risk of fatal outcome among mental disorders, have a tendency to chronicity, disability with social disadaptation, high suicidal risk. The psychopathological basis of these diseases is dysmorphophobia, characterized intrusive, overvalued or delusional ideas of physical disability. The significant role of dysmorphophobia determines the urgency of the detailed study using psychometric techniques.

**Objectives:**

Assess the degree of satisfaction/dissatisfaction with one’s body and its separate parts in patients with AN and BN.

**Methods:**

130 female patients with AN and BN at the age of 13-44 years (the average age is 18). The disease duration from 6 months to 24 years. The psychometric method using the validated Questionnaire image of one’s own body (QIOB) and the Scale of satisfaction with one’s body (SSOB).

**Results:**

According to QIOB 84,62% in the category expressed dissatisfaction with their appearance, 15,38% in moderate category. According to SSOB, 32,31% of the patients is not satisfied with characteristics that belong to head, 45,38% is not satisfied with characteristics that belong to torso, 56,92% is not satisfied with characteristics that belong to the lower part of body. The number of dissatisfied with all of these body parts equals 38% which indicates the presence of polydismorfofobia.

**Conclusions:**

High rates of dissatisfaction with one’s appearance, which are consistent with the severe somatic state of patients, affect the dynamics and outcome of the disease. Publication was prepared with support of the “RUDN University Program 5-100”.

